# Detection of mutations in the VP7 gene of vaccine-derived strains shed by monovalent rotavirus vaccine recipients

**DOI:** 10.1099/acmi.0.000033

**Published:** 2019-09-03

**Authors:** Mei Kaneko, Sayaka Takanashi, Mana Inoue, Hiroshi Sakiyama, Shoko Okitsu, Masashi Mizuguchi, Hiroshi Ushijima

**Affiliations:** ^1^​ Department of Developmental Medical Sciences, Graduate School of Medicine, The University of Tokyo, Bunkyo, Tokyo, Japan; ^2^​ Sakiyama Pediatric Clinic, Fuchu, Tokyo, Japan; ^3^​ Division of Microbiology, Department of Pathology and Microbiology, School of Medicine, Nihon University, Itabashi, Tokyo, Japan

**Keywords:** rotavirus, vaccine, shedding, VP7 gene, mutation

## Abstract

Strains of Rotarix, a live attenuated monovalent oral rotavirus vaccine, replicate in the intestine and are shed for about one month in immunocompetent recipients. The current study aimed to identify genetic changes of shed strains to reveal any significant mutations and their clinical impact on recipients. Stool samples of recipients of the first dose of Rotarix were sequentially collected for one month from the day of administration. Sequence analyses of the VP7 gene in eight recipients revealed five amino acid substitutions. Among them, two were observed in aa123, which is located in antigenic region 7-1a. Since there were no associated clinical symptoms, the genetic changes were unlikely to have caused reversion of pathogenicity of vaccine strain. Of interest, the virus in one case became closer to wild-type rotavirus via an amino acid change at aa123 occurring 14 days after administration, which might have resulted from multiple replications and long-term shedding of the vaccine strain.

## Introduction

Rotavirus infection is a leading cause of acute gastroenteritis (AGE) among children under five years of age [1]. Rotaviruses contain 11 segments of double-stranded RNA encoding six structural proteins, VP1–VP4, VP6 and VP7, and six nonstructural proteins, NSP1–NSP5/6 (2). VP7, along with VP4, is an outer capsid neutralizing antigen, and is critical for genotyping of group A rotaviruses. Rotarix (GlaxoSmithKline, Rixensart, Belgium) is a live rotavirus monovalent vaccine, which is attenuated by multiple tissue culture passages of human G1P[8] parental strain [2]. Two doses are orally administered to infants at intervals of more than four weeks before six months of age. Although the vaccines are highly effective against severe rotavirus gastroenteritis [2], several concerns need to be mentioned. The first is shedding of vaccine strain. Shedding is detected as early as one day and as late as 28 days [3] with documented cases of horizontal transmission [4]. In immunocompromised infants, shedding is prolonged, for instance, for more than six months in infants with severe combined immune deficiency (SCID) [5]. The second concern is genetic alterations in the shed vaccine strains. A few studies reported detection of mutations in VP7, VP4, VP6 and NSP4 genes of those shed strains [6, 7]. However, the mutations were not analysed in detail. The position and frequency of the mutations, and their relevance to antigenicity of viruses remain to be clarified.

This study aimed to identify nucleotide mutations in vaccine strain sequentially shed after vaccination of the first dose of Rotarix to reveal any significant genetic changes and their clinical impact on recipients.

## Methods

Forty healthy infants vaccinated with the first dose of Rotarix at Sakiyama Pediatric Clinic in Tokyo were enrolled in the study from September 2014 to March 2015. Informed consent was obtained from guardians of all study participants. This study was approved by the ethical committee of the University of Tokyo under the registration number of 2014–10528.

Stool samples were collected sequentially from the day of vaccination (day 0) to day 14, followed by day 21 and day 28 with health condition record. Viral titres of shed Rotarix strains had been quantified by real-time reverse transcription (RT)-PCR essentially according to the method descried by Gautam *et al*. [8]. Briefly, after RNA extraction by QIAamp Viral RNA mini kit (QIAGEN, Hilden, Germany), the viral titre was determined using TaqMan Fast Virus 1-Step Master Mix (Applied Biosystems, Foster City, CA, USA), Rotarix-specific primers (NSP2-F and NSP2-R), and probe (NSP2-P) with a standard curve generated from serial dilutions of plasmid DNA carrying targeted region of Rotarix NSP2 (nt 592–872; 281 bp). The samples that met the following criteria were further analysed in this study: (i) samples from infants with four time points: one between day 1 and day 3, one between day 12 and day 14, one from day 21 and one from day 28; (ii) viral titre of Rotarix strain was more than 10^6^ copies/g of stool; (iii) VP7 gene amplified fully. Samples from 29 infants met the criteria of (i) and samples from 11 infants further met the criteria of (ii). Samples from eight infants finally met the criteria of (iii). Consequently, 32 samples from eight infants were available for analysis in the current study.

Sanger sequencing for the VP7 gene was performed essentially according to the method descried by Gouvea *et al*. [9] and Fujii *et al*. [10]. Briefly, after denaturation of extracted double-stranded rotavirus RNA with 50 % (v/v) dimethyl sulfoxide, RT reaction was performed with SuperScript III Reverse Transcriptase (Invitrogen, Carlsbad, CA, USA). Synthesized cDNA was then used for PCR with PrimeSTAR GXL DNA polymerase (Takara, Shiga, Japan) with VP7 specific primers, Beg9 and End9. If the first attempt of amplification of VP7 gene failed, PCR incorporating inner primers Mid1r and Mid2 described by Trinh *et al*. [11] was performed to get overlapping fragments of the VP7 gene.

To confirm the important nucleotide mismatches between current samples and Rotarix original strain in antigenic region 7-1a, TA cloning was performed for a set of representative infants. Partial VP7 gene (nt 373–662; 290 bp) was cloned using TA Cloning Kit (Invitrogen, Carlsbad, CA, USA), pCR 2.1 vector (Invitrogen, Carlsbad, CA, USA) and Competent *Escherichiacoli* DH5αcells (Nippon Gene, Tokyo, Japan). In total, five white clones of each sample were picked up and incubated with vigorous shaking in liquid LB (Lysogeny broth) culture at 37℃ overnight. Plasmid DNA was extracted using AxyPrep Plasmid Miniprep Kit (Funakoshi, Tokyo, Japan). The nucleotide sequences of the inserted PCR amplicons were determined by the universal M13-Forward primer.

## Results and discussion

The age of eight infants and the details of each sample were described in Table 1. All the sequences were aligned with the VP7 gene of Rotarix original strain (accession no. JX943614). In total, ten nucleotide mutations were identified in six infants. Half of these mutations were nonsynonymous substitutions in amino acid at the position of aa 123, aa 134, aa 151 and aa 280 of the VP7 gene (Tables 2 and 3). The rate of mutation ranged from 0.00 to 0.21 %, and that of substitution from 0.00 to 0.63 %, respectively. Among five substitutions, two were found at the same position of aa 123, which belongs to antigenic region 7-1a as defined previously [13]. The substitutions at aa 123 were detected in two infants, no.9 and no.19. In infant no.9, both serine (S) and asparagine (N) were detected on day 3. In infant no.19, an amino acid substitution from S to N was detected since day 14 and it lasted until day 28.

**Table 1.** Age of infants and details of each sample

Infant no.	Age^*a*^ (week)	Day of sample collection and viral titre (log 10 copies/g of stool)			
		Day (viral titre)	Day (viral titre)	Day (viral titre)	Day (viral titre)
**2**	10	1 (7.81)	14 (7.72)	21 (6.90)	28 (6.72)
**6**	10	2 (9.08)	14 (9.89)	21 (9.94)	28 (6.77)
**9**	14	3 (9.31)	14 (9.88)	21 (9.90)	28 (9.43)
**10**	11	1 (7.62)	14 (8.14)	21 (7.08)	28 (7.68)
**17**	8	1 (8.70)	14 (9.30)	21 (7.43)	28 (8.21)
**19**	9	1 (8.13)	14 (7.93)	21 (9.08)	28 (8.86)
**22**	10	1 (8.58)	14 (8.88)	21 (7.50)	28 (7.70)
**35**	9	3 (8.88)	13 (8.79)	21 (8.20)	28 (7.97)

*a*, Age at vaccination of the first dose of Rotarix.

**Table 2.** Number and rate of mutations and substitutions detected among 32 samples from 8 infants

Infant no.	Mutations of nucleotide			Substitutions of amino acid		
	Sequenced length^*a*^ (bp)	Total no. of mutations	Mutation rate^*b*^ (%)	Sequenced length^*c*^ (aa)	Total no. of substitutions	Substitution rate^*d*^ (%)
**2**	957	2	0.21	319	2	0.63
**6**	924	0	0.00	309	0	0.00
**9**	981	2	0.20	326	1	0.31
**10**	947	2	0.21	317	1	0.32
**17**	933	1	0.11	311	0	0.00
**19**	940	2	0.21	308	1	0.32
**22**	990	0	0.00	326	0	0.00
**35**	1001	1	0.10	326	0	0.00
**Average**	959	1.25	0.13	318	0.63	0.20

*a*, Whole nucleotide length of the VP7 gene is 1062 bp.

*b*, Mutation rate was calculated as the total number of mutations/sequenced length (bp).

*c*, Whole amino acid length of the VP7 gene is 326 aa.

*d*, Substitution rate was calculated as the total number of substitutions/sequenced length (aa).

**Table 3.** Position and timing of the mutations and substitutions

Infant no.	Mutations of nucleotide						Substitutions of amino acid						
	Position (nt)	Day1–3	Day14	Day21	Day28	Rotarix^*a*^	Position (aa)	Day1–3	Day14	Day21	Day28	Rotarix^*a*^	Antigenic region^*c*^
**2**	nt 499	G	G	G	**A**	G	aa 151	D	D	D	**N**	D	-
	nt 887	A	A	A	**G**	A	aa 280	Q	Q	Q	**R**	Q	-
**9**	nt 321	**T / C** ^***b***^	T	T	T	T	aa 91	T	T	T	T	T	7–1 a
	nt 416	**G / A** ^***b***^	G	G	G	G	aa 123	**S / N**	S	S	S	S	7–1 a
**10**	nt 448	T	T	**T / C** ^***b***^	T	T	aa 134	Y	Y	**Y / H**	Y	Y	-
	nt 834	A	A	**G**	A	A	aa 262	Q	Q	Q	Q	Q	-
**17**	nt 445	T	**C**	**C**	**C**	T	aa 133	L	L	L	L	L	-
**19**	nt 316	**G**	A	A	A	A	aa 90	S	S	S	S	S	-
	nt 416	G	**A**	**A**	**A**	G	aa 123	S	**N**	**N**	**N**	S	7–1 a
**35**	nt 981	C	T	T	T	C	aa 311	S	S	S	S	S	-

Nucleotides and amino acids covered by shadowing indicate mutations or substitutions compared with those of the Rotarix original strain.

*a*, Sequence of the VP7 gene of the Rotarix original strain was based on data deposited in GenBank (JX943614) [8].

*b*, Two peaks with the same height were identified in nucleotide sequences.

*c*, The definition of antigenic regions is based on the previous study [12].

TA cloning was carried out for the four samples from infant no.19 (samples of day 1, day 14, day 21 and day 28) to investigate the mutations and substitutions detected at aa 123. The TA cloning was also performed on the original Rotarix solution (GlaxoSmithKline, Rixensart, Belgium; Lot: AROLB224AA) to confirm the existence of the nucleotides at aa 123, though it is deposited as S in GenBank with accession no. JX943614. Ratio of nucleotide (amino acid) at nt 416 (aa 123) in each sample was summarized in Table 4. Based on the results of TA cloning, the substitutions were observed from day 14 to day 28 as expected, with all the clones possessing N instead of S. Four out of five clones of the Rotarix original strain possessed S at aa 123 in accordance with the amino acid deposited in GenBank. Interestingly, one clone was found to possess N.

**Table 4.** Ratio of nucleotide (amino acid) at nt 416 (aa 123) in each sample

Nucleotide at nt 416	Amino acid at aa 123	Day 1	Day 14	Day 21	Day 28	Rotarix^*a*^
**G**	**Serine (S**)	**5**	0	0	0	**4**
**A**	**Asparagine (N**)	0	**5**	**5**	**5**	**1**
**Total no. of clones**		5	5	5	5	5

*a*, Rotarix original solution (GlaxoSmithKine, Rixensart, Belgium; Lot:AROLB224AA) was used.

Ten nucleotide mutations and five amino acid substitutions in the VP7 gene of shed Rotarix strain were detected among vaccine recipients. The average rate of the mutations in the VP7 gene was 0.13 % within 28 days(Table 2). Based on this result, the evolutionary rate of the Rotarix VP7 gene was calculated as 1.00×10^−4^ nucleotide substitutions per site per year (s/s/y). As proven, RNA viruses mutate quickly and hence evolve rapidly because of their error prone replications [14]. For several RNA viruses including rotaviruses, overall evolutionary rates range from 10^−2^ to 10^−5^ nucleotide s/s/y. In particular for rotaviruses, the evolutionary rate of the VP7 gene was 7.25×10^−4^ nucleotide s/s/y [15]. Taken together, the evolutionary rate detected in this study was comparable to those reported in previous studies.

Based on the results of TA cloning, the strain of the orignal Rotarix solution was found to possess both S and N at aa 123, with predominance of S. The coexistence of two amino acids at the same position was likely due to quasispecies of RNA viruses [16]. Multiple cell passages for attenuation of the parental strain of Rotarix might be responsible for the mutations and quasispecies.

The aa 123 is located in antigenic region 7-1a that spans the intersubunit boundary [14, 17]. The genetic changes occur at comparatively higher frequency in this region. However, the substitution at aa 123 reportedly does not permit escape from neutralization, as demonstrated using various monoclonal antibodies [15]. Therefore, it may not be responsible for antigenic alteration in the mutated strain. Of note, several studies on amino acid differences in VP7 and VP4 antigenic sites between wild-type rotaviruses and vaccine strains reported that most of the circulating wild-type strains possessed N instead of S at aa 123 [17, 18]. Therefore, we inferred that the amino acid at aa 123 was likely to have changed the strain closer to that of wild-type strains through multiple replications in intestine during the shedding periods.

Importantly, the shedding of the Rotarix strain in eight infants was not associated with any symptoms of gastroenteritis. Therefore, the changes of nucleotide and amino acid detected in this study neither produced clinical symptoms nor reversed pathogenicity of Rotarix strain.

Further investigations are necessary not only for healthy infants, but also for those with impaired immunity suffering from severe illnesses associated with vaccine strains. In particular, vaccine or vaccine-derived strains detected in infants with SCID should be assessed in detail since the strains would be shed with a high titre and for a long period, resulting in severe and persisting gastroenteritis. To clarify the evolution of vaccine strains and the clinical impact of the mutants on infants, the other remaining ten genes should be analysed as well. In particular, VP4 and NSP4, which encode another neutralizing antigen and enterotoxin respectively, could be significant targets [19]. Analyses of these key genes might provide the clues to identify the mutations related to pathogenicity of the vaccine strains.
